# Esophageal squamous cell carcinoma complicated with varices successfully treated by endoscopic injection sclerotherapy and argon plasma coagulation: A case report

**DOI:** 10.1002/deo2.348

**Published:** 2024-02-28

**Authors:** Sayuri Watanabe, Takuto Hikichi, Takumi Yanagita, Jun Nakamura, Minami Hashimoto, Tsunetaka Kato, Ryoichiro Kobashi, Yuichi Waragai, Masao Kobayakawa, Hiromasa Ohira

**Affiliations:** ^1^ Department of Gastroenterology Fukushima Medical University School of Medicine Fukushima Japan; ^2^ Department of Endoscopy Fukushima Medical University Hospital Fukushima Japan; ^3^ Department of Gastroenterology Soma General Hospital Fukushima Japan; ^4^ Medical Research Center Fukushima Medical University Fukushima Japan

**Keywords:** argon plasma coagulation, endoscopic injection sclerotherapy, endoscopic submucosal dissection, esophageal squamous cell carcinoma, esophageal varices

## Abstract

Treatment guidelines for esophageal squamous cell carcinoma (ESCC) with concomitant esophageal varices (EVs), which increase the risk of bleeding, are unavailable. A 66‐year‐old man with a history of total gastrectomy was admitted to the hospital owing to hematemesis. Emergency upper gastrointestinal endoscopy revealed variceal bleeding near the anastomosis between the esophagus and jejunum, and endoscopic clipping stopped the bleeding. Upper gastrointestinal endoscopy following hemostasis revealed four EVs and a two‐thirds ESCC circumference. The ESCC depth was suspected to be up to the mucosa. The patient underwent intravariceal endoscopic injection sclerotherapy (EIS) for EVs, followed by paravariceal EIS. However, after these treatments, blood flow in the EVs just below the ESCC remained, and endoscopic resection of the ESCC was judged to be difficult to perform. Therefore, we prioritized EV treatment and performed a second EIS on the ESCC, followed by argon plasma coagulation (APC). APC was expected to not only solidify the EVs but also eliminate the ESCC existing in the mucosa. Finally, EVs and ESCC were treated by EIS and APC. EIS followed by APC may be useful for treating concurrent EVs and intramucosal ESCC in patients with liver cirrhosis when embolization of the EVs is ineffective.

## INTRODUCTION

Heavy alcohol consumption is one of the risk factors for esophageal squamous cell carcinoma (ESCC). It can cause liver cirrhosis (LC) and esophageal varices (EV). The standard treatment for ESCC with a low risk of lymph node metastasis is endoscopic resection; endoscopic submucosal dissection (ESD) is becoming popular, particularly in Asia. Conversely, 0.8%–1.6% of patients with ESCC who underwent ESD had EVs.^1^, ^2^ EV treatment before ESD causes submucosal fibrosis in the area of ESCC, making it difficult to dissect the submucosa and creating a risk of perforation during ESD. Although ESD for ESCC complicated with EV has been reported to be safe and effective in several retrospective studies,^1^, ^2^ other studies have reported a potential risk of critical bleeding or perforation.^3^, ^4^

In this report, we describe a case of EVs and ESCC successfully treated by endoscopic injection sclerotherapy (EIS), followed by argon plasma coagulation (APC).

## CASE REPORT

A 66‐year‐old male presented to our hospital for hematemesis; he had a history of total gastrectomy with Roux‐en‐Y reconstruction and splenectomy for gastric cancer at the age of 55 years. Upper gastrointestinal (UGI) endoscopy had not been performed for 11 years after the surgery. Computed tomography (CT) was last performed 6 years after surgery. LC and EVs were undetected at the time of the surgery, and the presence of these diseases has not been noted thereafter. He had consumed 80 g ethanol equivalent alcohol daily. Blood tests revealed hemoglobin, albumin, and total bilirubin levels of 11.0 g/dL, 3.1 g/dL, and 2.0 mg/dL, respectively, and negative hepatitis B virus surface antigen and hepatitis C virus antibody.

**FIGURE 1:**
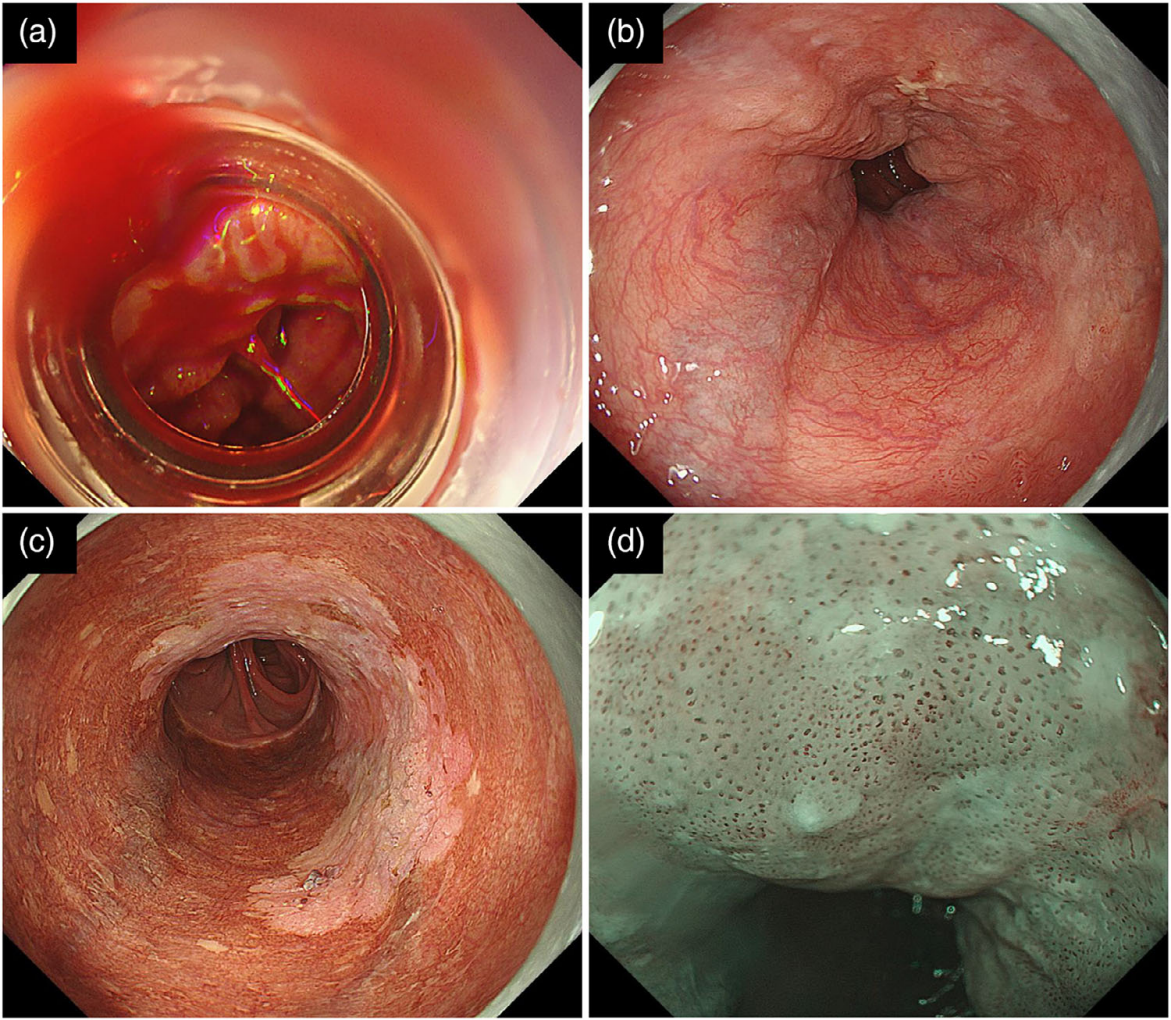
Endoscopic findings at the time of bleeding esophageal varices and esophageal squamous cell carcinoma diagnosis. (a) An eruptive hemorrhage is observed at the esophageal jejunal anastomosis. Endoscopic variceal ligation is first attempted. However, the varices could not be aspirated owing to postoperative scarring, and hemostasis could not be achieved. Finally, endoclips have stopped variceal bleeding. (b) Endoscopic examination following hemostasis reveals four varices in the esophagus. (c) A 2/3 circumference of the iodine‐unstained area is observed. Biopsy from the lesion revealed squamous cell carcinoma. In addition, three of the four varices are located just below the squamous cell carcinoma. (d) Magnification endoscopy with narrow‐band imaging shows dilatation of the intrapapillary capillary loops corresponding to the Japanese Esophageal Society's B‐1 vessels. The lesion depth is considered to be less than lamina propria mucosae.

UGI endoscopy revealed bleeding near the anastomosis between the esophagus and jejunum (Figure 1a). Endoscopic variceal ligation (EVL) failed to stop bleeding; however, endoscopic clipping was successfully performed. UGI endoscopy 4 days following the first procedure revealed four linear EVs: LmF1CbRC0 based on the classification of the Japan Society for Portal Hypertension^5^ (Figure 1b). Additionally, a two‐thirds circumferential ESCC was observed, extending from near the anastomosis to the oral side (Figure 1c). Regarding ESCC diagnosis, there was no prominent bulge or depression, and narrow‐band imaging magnifying endoscopy revealed a cluster of B‐1 vessels based on the classification of the Japanese Esophageal Society^6^ (Figure 1d). Therefore, we diagnosed the depth of ESCC to be within the lamina propria mucosae (LPM). Three of the four EVs were located just below the ESCC. CT revealed no evidence of lymph node or distant metastasis, and the patient was diagnosed with clinical Stage 0 (cT1aN0M0). Moreover, he was diagnosed with Child–Pugh class B alcoholic LC.

**FIGURE 2:**
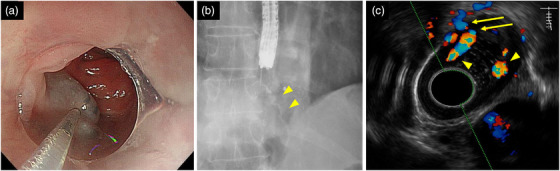
Endoscopic findings during and after endoscopic injection sclerotherapy. (a) Image showing 0.4‐mL ethanolamine oleate is injected into the varix near the esophageal jejunal anastomosis on the anorectal side of the esophageal squamous cell carcinoma. (b) Ethanolamine oleate injection from the varix into the blood supply routes is confirmed by X‐ray contrast image (yellow arrowheads). (c) Two months following the first intravariceal endoscopic injection sclerotherapy and paravariceal endoscopic injection scerlotherapy, residual varices (yellow arrowheads) and a penetrating vein (yellow arrows) are observed just below the esophageal squamous cell carcinoma on endoscopic ultrasonography.

We considered that ESD for ESCC without prior treatment of EVs would result in critical bleeding, and preferred EIS over EVL for EV treatment to avoid submucosal fibrosis. Therefore, we decided to perform intravariceal EIS by injecting ethanolamine oleate (EO) into the EVs on the anorectal side of the ESCC. We planned to perform ESD for ESCC following confirmation of decreased blood flow in the EVs by endoscopic ultrasonography (EUS). A single‐channel endoscope and a 25‐G puncture needle (Varixar; Top Co.) were used to perform intravariceal EIS under fluoroscopy. As a sclerosant, 5% EOI combined with 10% EO (Oldamine; Fuji Chemical Industry) and the same amount of contrast agent were used (Figure 2a,b). One week following intravariceal EIS, as EV blood flow remained around the ESCC, paravariceal EIS with 1% polidocanol (Aethoxysklerol [AS]; Kaigen Pharma) was performed. While avoiding the ESCC, AS was injected near the anastomosis.

**FIGURE 3:**
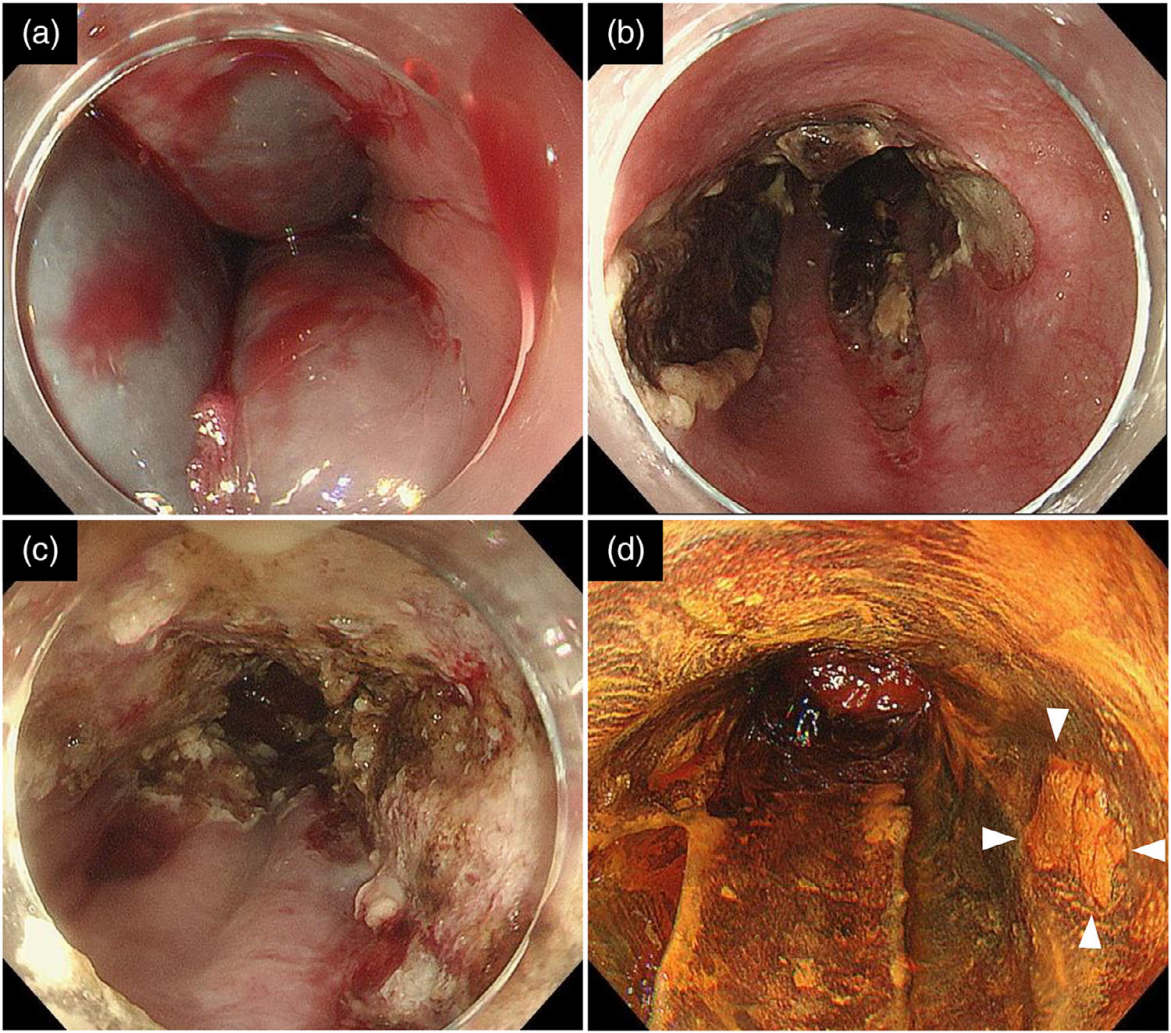
Argon plasma coagulation (APC) for esophageal varices and esophageal squamous cell carcinoma. (a) In a second session of paravariceal endoscopic injection scleropathy performed 1 week prior to APC, marked hematomas developed during paravariceal endoscopic injection sclerotherapy; in total, 30 mL of AS was injected. (b) Endoscopic findings immediately before APC showed a prominent ulcer after paravariceal endoscopic injection scleropathy. Therefore, APC was performed to avoid the ulcers. (c) APC is performed at a 1.2‐L/min flow rate and a 30‐W radiofrequency power in the esophageal squamous cell carcinoma area and 5 cm proximally from the esophageal jejunal anastomosis. (d) Four weeks following APC, endoscopy reveals a remnant of localized esophageal squamous cell carcinoma (yellow arrowhead), and additional APC is performed.

**FIGURE 4:**
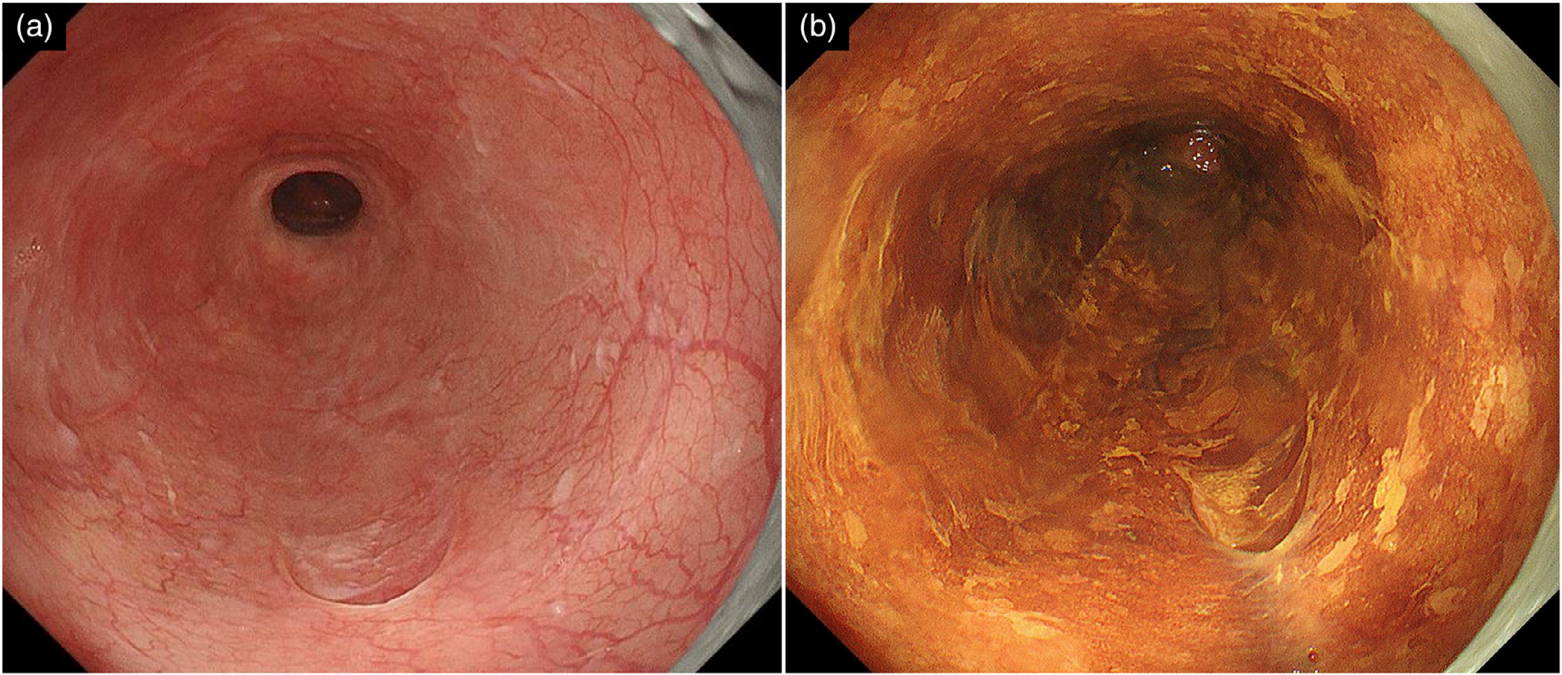
Endoscopic findings following 1 year of argon plasma coagulation. (a) The esophageal mucosa is scarred, and no recurrence of esophageal varices is observed. (b) No iodine staining suggestive of esophageal squamous cell carcinoma is noted. Therefore, a biopsy was not performed.

Two months following the EIS, UGI endoscopy and EUS still revealed EVs and perforating veins just below the ESCC (Figure 2c). Subsequently, chemoradiotherapy or surgical esophagectomy was considered as an alternative treatment for ESCC. However, concerns about the risk of jejunal perforation by radiation and complications due to invasive surgery in patients with LC were noted. Therefore, additional EIS on the ESCC and APC were selected for EV and ESCC treatment. We expected that these treatments could simultaneously eliminate ESCC. Intravariceal EIS with 5% EOI injection into the EVs just below the ESCC was performed, and paravariceal EIS with AS was performed 1 week after the intravariceal EIS. However, during the paravariceal EIS, hematomas unexpectedly formed along the EV morphology. One week following paravariceal EIS, APC was performed using VIO3 (ERBE Elektromedizin) and an APC probe. After the paravariceal EIS, avoiding the ulcerated areas caused by the hematoma, APC (setting: forced APC, effect 3, 1.2 L/min) was performed from the anastomosis of the esophagus and jejunum to 5 cm proximally as consolidation therapy for EVs, and APC was performed to eliminate the ESCC (Figure 3a). Since the ESCC was located within this area, we judged that ablation of the tumor could also be performed. No procedure‐related adverse events occurred. Four weeks following APC, the EVs disappeared; however, localized residual ESCC was observed on endoscopy, and additional APC was performed (Figure 3b). Two months following the additional APC, UGI endoscopy confirmed no residual ESCC. One year following APC, no EV or ESCC recurrence was observed (Figure 4a,b).

## DISCUSSION

This paper reports a case with a history of total gastrectomy wherein EIS and subsequent APC were effective for treating ESCC and EV at the same site.

In the present case, the ESCC depth was diagnosed endoscopically within the LPM; therefore, the lesion was considered to be indicated for ESD. The presence of EVs not only around the ESCC but also in the submucosa just below the ESCC was a serious issue. The success of ESD for ESCC complicated by EVs depends on the strategy employed for preventing bleeding from the EVs. Some retrospective studies^1^, ^2^ have reported safe ESD for ESCC complicated with EVs; however, cases wherein ESD was abandoned midway owing to bleeding from the EVs have also been reported.^3^, ^4^ In one case, despite performing intravariceal and paravariceal EIS and avoiding ESCC, considerable blood flow remained in the EVs, and submucosal fibrosis made submucosal dissection challenging, thereby resulting in ESD discontinuation.^3^ Although there was a report wherein EVL was successfully performed on the anorectal side of ESCC to control EV blood flow,^4^ EVL would not have been suitable for our case because the jejunum would be involved in ligation. Furthermore, the perforating vein just below the ESCC made it difficult to treat the EV without affecting the ESCC.

APC is recommended as an additional EV treatment for preventing recurrence following EIS or EVL.^7^ APC cauterizes the esophageal mucosa with radiofrequency coagulation, thereby resulting in ulceration of the entire esophagus. Mucosal and submucosal fibrosis occurs throughout the healing process of the ulcer created by APC; therefore, EVs do not form even if blood flow reenters the esophagus.

Regarding APC applied to the treatment of ESCC, local recurrence rates of 2.5%–20% have been reported.^8^, ^9^ Kawada et al. ^10^ reported that intramucosal ESCC lesions could be controllable by APC. As ESD, unlike APC, can be used to evaluate the depth of lesion pathologically, it is the first choice for ESCC treatment. However, as in this case, because the blood flow within the EVs could not be controlled, we concluded that ESD posed a high risk of intraoperative bleeding. Additionally, since the depth of the lesion would be up to the LPM, we considered that APC could be effective. Therefore, we believe that APC may be an option for ESCC that is evaluated endoscopically intramucosal and difficult to be safely treated with ESD, as in this case. However, APC differs from ESD and surgical esophagectomy, it does not allow the histopathological evaluation of ESCC and careful follow‐up is necessary.

In summary, our results suggest that EIS followed by APC can be useful for treating concurrent EV and intramucosal ESCC in patients with LC when embolization of the EVs is ineffective.

## CONFLICT OF INTEREST STATEMENT

None.

## Data Availability

We would like to provide data as needed.
